# Association of airborne particles, protein, and endotoxin with emergency department visits for asthma in Kyoto, Japan

**DOI:** 10.1186/s12199-018-0731-2

**Published:** 2018-08-28

**Authors:** Mohammad Shahriar Khan, Souleymane Coulibaly, Takahiro Matsumoto, Yoshitaka Yano, Makoto Miura, Yukio Nagasaka, Masayuki Shima, Nobuyuki Yamagishi, Keiji Wakabayashi, Tetsushi Watanabe

**Affiliations:** 1 Department of Public Health, Kyoto Pharmaceutical University, 1 Misasagi-Shichonocho, Yamashinaku, Kyoto, 607-8412 Japan; 2 Education and Research Center for Clinical Pharmacy, Kyoto Pharmaceutical University, 5 Misasagi-Nakauchcho, Yamashinaku, Kyoto, 607-8414 Japan; 3 Rakuwakai Otowa Hospital, 2 Otowachinji-cho, Yamashinaku, Kyoto, 607-8062 Japan; 4 Department of Public Health, Hyogo College of Medicine, 1-1 Mukogawacho, Nishinomiya, Hyogo 663-8501 Japan; 5 Faculty of Pharmaceutical Sciences, Setsunan University, 45-1 Nagaotogecho, Hirakata, Osaka 573-0101 Japan; 6 Graduate Division of Nutritional and Environmental Sciences, University of Shizuoka, 52–1 Yada, Suruga-ku, Shizuoka, 422-8526 Japan

**Keywords:** Air pollution, Bioaerosols, Lipopolysaccharide, PM_2.5_, Coarse particle

## Abstract

**Background:**

The health effects of biological aerosols on the respiratory system are unclear. The purpose of this study was to clarify the association of airborne particle, protein, and endotoxin with emergency department visits for asthma in Kyoto City, Japan.

**Methods:**

We collected data on emergency department visits at a hospital in Kyoto from September 2014 to May 2016. Fine (aerodynamic diameter ≤ 2.5 μm) and coarse (≥ 2.5 μm) particles were collected in Kyoto, and protein and endotoxin levels were analyzed. The association of the levels of particles, protein, endotoxin, and meteorological factors (temperature, relative humidity, wind speed, and air pressure) with emergency department visits for asthma was estimated.

**Results:**

There were 1 to 15 emergency department visits for asthma per week, and the numbers of visits increased in the autumn and spring, namely many weeks in September, October, and April. Weekly concentration of protein in fine particles was markedly higher than that in coarse particles, and protein concentration in fine particles was high in spring months. Weekly endotoxin concentrations in fine and coarse particles were high in autumn months, including September 2014 and 2015. Even after adjusting for meteorological factors, the concentrations of coarse particles and endotoxin in both particles were significant factors on emergency department visits for asthma.

**Conclusions:**

Our results suggest that atmospheric coarse particles and endotoxin are significantly associated with an increased risk of asthma exacerbation.

**Electronic supplementary material:**

The online version of this article (10.1186/s12199-018-0731-2) contains supplementary material, which is available to authorized users.

## Background

Many epidemiological studies have shown that exposure to airborne particles is associated with the exacerbation of asthmatic symptoms and the increase of asthma-related emergency department visits [1–3]. However, other studies have reported contradictory results [4, 5]. Airborne particles are a mixture of diverse materials; this variation may cause differences in the health effects. Biological aerosols, or bioaerosols, are suspended airborne particles comprising microorganisms such as bacteria and fungi and organic materials originating from living organisms. For instance, allergic proteins originating from fungi and pollen have been detected in airborne particles [6, 7]. Total protein concentration is often used as an all-inclusive indicator of airborne biological material that may enhance allergic and asthmatic responses in aerobiological studies [8–10]. Endotoxins or lipopolysaccharides are the major components of the outer membrane of Gram-negative bacteria and have been detected in both fine (aerodynamic diameter ≤ 2.5 μm; PM_2.5_) and coarse (aerodynamic diameter ≥ 2.5 μm) particles collected in urban and rural regions [11–14]. The inhalation of endotoxins stimulates the alveolar macrophages and respiratory epithelial tissue to release cytokines or chemoattractants that initiate an inflammatory cascade [15]. Epidemiological and controlled exposure studies have indicated that endotoxin exposure is positively associated with the exacerbation of asthma [16, 17]. As described above, airborne particles, protein, and endotoxin may cause the exacerbation of asthma; therefore, knowing the concentrations of these air pollutants is important for patients with asthma to avoid exacerbation of their symptoms. However, there are few reports on the long-term levels of proteins and endotoxins in the outdoor air of Japan [14].

The purpose of this study was to measure the concentrations of airborne particles, protein, and endotoxin in outdoor air and their associations with asthma. We collected fine (aerodynamic diameter ≤ 2.5 μm) and coarse (≥ 2.5 μm) particles and data on emergency department visits for asthma at a hospital in Kyoto City, Japan, from September 2014 to May 2016 and analyzed proteins and endotoxin levels in both types of particle. Kyoto is a core city of the Keihanshin metropolitan area, which is the second largest metropolitan area of Japan. The population of Kyoto City is 1.5 million, and its key industries are information technology, electronics, and tourism. Because meteorological factors such as temperature and relative humidity are reportedly associated with emergency department visits for asthma [18, 19], we used regression analysis to investigate the association of the levels of particles, protein, endotoxin, temperature, relative humidity, wind speed, and air pressure with emergency department visits for asthma.

## Methods

### Data on emergency department visits

We obtained anonymized data on emergency department visits for asthma at the Rakuwakai Emergency and Critical Care Center in Kyoto from September 2014 to May 2016. The data included information on patient age, diagnosis, and date of visit. The patients in this study were adults (aged 15 years or older) and children (< 15 years). This retrospective observational study was approved by the ethics committee of Kyoto Pharmaceutical University (Approval No. 17-16-18) and Rakuwakai Otowa Hospital (Approval No. 16-018).

### Environmental data

Fine and coarse particles were collected on glass filters (Advantec Co., Ltd., Tokyo, Japan) and quartz filters (Pall Life Sciences, Port Washington, NY, USA), respectively, in Kyoto (135.81° E, 34.99° N) using a high-volume air sampler (HV1000R; Shibata Scientific Technology, Soka, Japan) equipped with an impactor (Shibata Scientific Technology) at a flow rate of 1 m^3^/min for 1 week per filter. The filters were heated at 250 °C for 2 h prior to use. Particle collection was performed for 2 to 4 weeks per month from September 2014 to May 2016 (Table 1). In total, 154 samples (77 sets each of fine and coarse particles) were collected. In October 1–14, 2014, we could not collect samples for seven consecutive days because of a typhoon. We eliminated 3 weeks, namely April 30 to May 6 and September 18–24, 2015, and May 2–8, 2016, because these weeks included three or four national holidays, and the number of emergency department visits for asthma might have increased in these weeks. The filters were weighed before and after the collection of airborne particles. After the sample collection, the filters were kept in a freezer at − 80 °C until protein and endotoxin measurement.

**Table 1:** Sampling periods of the particles

Year	Month	Week
2014	September	1st (Sep. 1–7), 2nd (8–14), 3rd (15–21), 4th (22–28)
	October	1st (Oct. 15–21), 2nd (22–28)
	November	1st (Oct. 31–Nov. 6), 2nd (7–13), 3rd (14–20), 4th (21–27)
	December	1st (Dec. 1–7), 2nd (8–14), 3rd (15–21), 4th (22–28)
2015	January	1st (Jan. 6–12), 2nd (13–19), 3rd (20–26)
	February	1st (Feb. 2–8), 2nd (9–15), 3rd (16–22), *4th* (*Feb*. *23*–*Mar*. *1*)
	March	1st (Mar. 2–8), 2nd (9–15), *3rd* (*16*–*22*), 4th (23–29)
	April	1st (Apr. 2–8), 2nd (9–15), 3rd (16–22), 4th (23–29)
	May	1st (May 7–13), 2nd (15–21th), 3rd (22nd–28th)
	June	1st (Jun. 1–7), *2nd* (*8*–*14*), 3rd (15–21), 4th (22–28)
	July	1st (Jul. 3–9), 2nd (10–16), 3rd (17–23), 4th (24–30)
	August	1st (Aug. 3–9), 2nd (10–16), 3rd (17–23), 4th (24–30)
	September	1st (Sep. 4–10), 2nd (11–17), 3rd (Sep. 25–Oct. 1)
	October	1st (Oct. 2–8), 2nd (9–15), 3rd (16–22), 4th (23–29)
	November	1st (Nov. 4–10), 2nd (11–17), 3rd (18–24), 4th (Nov. 25–Dec. 1)
	December	1st (Dec. 2–8), 2nd (9–15), 3rd (16–22)
2016	January	1st (Jan. 6–12), 2nd (13–19), 3rd (20–26), 4th (Jan. 27–Feb. 2)
	February	1st (Feb. 3–9), 2nd (10–16), 3rd (17–23), 4th (Feb. 24–Mar. 1)
	March	1st (Mar. 3–9), 2nd (10–16), 3rd (17–23), 4th (24–30)
	April	1st (Apr. 1–7), 2nd (8–14), 3rd (15–21), *4th* (*22*–*28*)
	May	1st (May 9–15), 2nd (16–22), 3rd (23–29)

The Asian dust event was observed in Kyoto by Japan Meteorological Agency on the following days: Feb. 23 and 24, Mar. 22, and Jun. 13 in 2015, and Apr. 24 and 25 in 2016. The weeks written in italics are periods that Asian dust event was observed

To analyze protein and endotoxin levels, 15% of the sample filters (corresponding to 1512 m^3^ of air) were extracted using 0.025% Tween 20 for 30 min by an ultrasonic apparatus [14]. The extract was centrifuged, and a portion of the supernatant was used for the protein and endotoxin analyses.

The protein levels were analyzed using a bicinchoninic acid assay (Micro BCA Protein Assay Kit; Thermo Fisher Scientific, Rockford, IL, USA) according to the manufacturer’s protocols and a microplate reader (Sunrise Thermo RC-R; Tecan Austria GmbH, Groedig, Austria), as described previously [14]. The absorption of all 154 samples was higher than the detection limit (2.5 μg/mL), while that of a blank filter was lower than the detection limit.

The endotoxin levels were analyzed using the kinetic chromogenic Limulus amebocyte lysate (LAL) method (Limulus Color KY Test Wako kit; Wako Pure Chemical Industries, Ltd., Osaka, Japan) according to the manufacturer’s protocols with a microplate reader (Sunrise Thermo RC-R), as described previously [14]. Absorption of all 154 samples was higher than the detection limit (0.0005 EU/mL), while that of a blank filter was lower than the detection limit. For the spiked samples, the recovery rates were in the range of 50–200%, which were considered acceptable according to the instructions for the LAL assay kit.

Data on the daily mean ambient temperature, relative humidity, wind speed, and air pressure in Kyoto City were acquired from the Japan Meteorological Agency [20].

### Statistical analyses

Microsoft Office 2013 was used to calculate correlation coefficients. For the analyses of the association between the weekly numbers of emergency department visits and the weekly levels of environmental factors, we used the generalized linear models to fit a Poisson regression. The analyses were performed using SPSS Statistics Version 22 (IBM Software Group, Chicago, IL, USA). A *p* value of < 0.05 was considered statistically significant.

## Results

### Emergency room visits for asthma

**Fig. 1:**
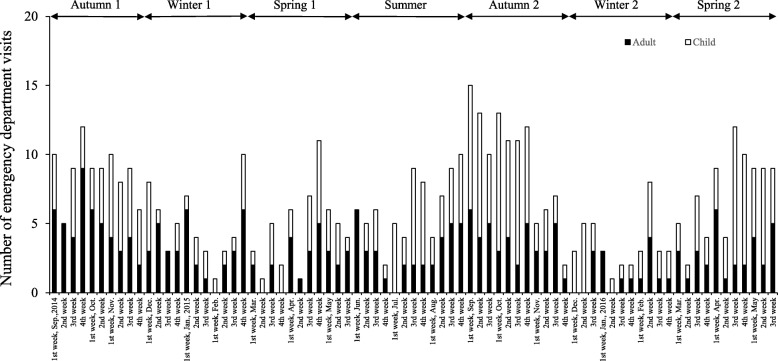
Number of emergency department visits for asthma at Rakuwakai Otowa Hospital in Kyoto. The study period (September 2014 to May 2016) was divided into autumn (September to November), winter (December to February), spring (March to May), and summer (June to August)

Figure 1 shows the weekly number of emergency department visits for asthma during the study period (September 2014 to May 2016). In total, there were 490 emergency department visits (229 adults and 261 children) during that period. The number of emergency department visits ranged from 1 to 15 per week, with a high number of visits in the autumn and spring months, namely September (fourth week) 2014, September (first and second weeks) and October (first and fourth weeks) 2015, and April (third week) 2016.

### Weekly levels of air pollutants, temperature, relative humidity, wind speed, and air pressure

The weekly concentrations of particles, protein, and endotoxin in the outdoor air of Kyoto City during the study period are shown in Additional file 1: Figures S1, S2, and S3, respectively. Protein and endotoxin were detected in all samples examined in this study. For the entire sampling period, the weekly concentration of fine particles was slightly higher than that of coarse particles; the fine and coarse particle concentrations were 6.2–32.3 and 2.4–23.8 μg/m^3^, respectively (Additional file 1: Figure S1). For fine particles, the atmospheric concentrations were high in spring months, including March and April 2015 and May 2016. The atmospheric mass concentrations of coarse particles were high in the fourth week of February and spring months, including April 2015. The weekly concentration of protein in fine particles was markedly higher than that in coarse particles throughout the entire sampling period; the weekly protein concentrations of fine and coarse particles were 0.17–5.09 and 0.02–0.46 μg/m^3^, respectively (Additional file 1: Figure S2). The concentrations of protein in fine particles were high in spring months, including April and May 2015 and May 2016. In contrast, weekly concentrations of endotoxin in coarse particles (0.0004–0.0292 EU/m^3^) were higher than those in fine particles (0.00003–0.01279 EU/m^3^), and the weekly concentrations of endotoxin in coarse particles were high in autumn months, including September 2014 and 2015 (Additional file 1: Figure S3).

Table 2 shows the whole and seasonal mean values and standard deviations of air pollutants and meteorological factors. The mean values and standard deviations were calculated with the weekly mean value of each factor. Mean values of fine and coarse particles and protein in fine particles were high in spring 1 and spring 2. In contrast, the mean value of endotoxin in coarse particles was high in autumn 1 and autumn 2. Mean value of temperature ranged from 5.6 °C (winter 1) to 26.1 °C (summer). Other meteorological factors did not show any noticeable fluctuation.

**Table 2:** Weekly mean value (standard deviation) of air pollutants and meteorological factors

	Fine particle			Coarse particle			Temperature(°C)	Relative humidity (%)	Wind speed (m/s)	Air pressure (hPa)
	Particles (μg/m^3^)	Protein (μg/m^3^)	Endotoxin (EU/m^3^)	Particles (μg/m^3^)	Protein (μg/m^3^)	Endotoxin (EU/m^3^)				
Whole	14.4 (5.2)	1.42 (0.84)	0.0019 (0.0022)	7.6 (3.3)	0.12 (0.08)	0.0052 (0.0052)	15.1 (7.7)	65.5 (6.4)	2.1 (0.3)	1010.2 (4.9)
Autumn 1	12.2 (2.1)	1.83 (0.74)	0.0031 (0.0035)	7.7 (1.1)	0.17 (0.11)	0.0098 (0.0089)	18.0 (5.2)	65.9 (5.0)	1.9 (0.26)	1010.7 (4.15)
Winter 1	13.2 (4.4)	1.26 (0.34)	0.0007 (0.0006)	7.1 (5.8)	0.07 (0.04)	0.0014 (0.0007)	5.6 (1.3)	69.2 (3.5)	2.0 (0.3)	1012.6 (2.7)
Spring 1	18.7 (7.4)	1.96 (0.70)	0.0020 (0.0022)	8.5 (3.3)	0.16 (0.05)	0.0039 (0.0026)	14.6 (5.6)	61.5 (9.2)	2.2 (0.2)	1010.0 (4.5)
Summer	12.7 (4.3)	0.79 (0.33)	0.0008 (0.0009)	8.1 (2.0)	0.19 (0.07)	0.0076 (0.0047)	26.1 (3.1)	68.6 (5.1)	2.2 (0.4)	1002.9 (1.8)
Autumn 2	11.3 (4.2)	0.95 (0.42)	0.0026 (0.0021)	7.4 (2.9)	0.05 (0.02)	0.0074 (0.0050)	18.1 (4.0)	67.1 (6.2)	1.9 (0.3)	1011.2 (4.2)
Winter 2	12.3 (3.1)	1.20 (0.71)	0.0010 (0.0005)	5.5 (2.1)	0.05 (0.03)	0.0017 (0.0004)	6.9 (2.6)	65.1 (4.4)	2.1 (0.3)	1014.4 (3.0)
Spring 2	17.8 (5.0)	2.05 (1.32)	0.0032 (0.0025)	8.6 (3.6)	0.12 (0.05)	0.0049 (0.0031)	15.5 (5.04)	61.0 (5.3)	2.2 (0.2)	1010.1 (3.7)

Table 3 shows the correlation coefficients for the weekly levels of airborne particles, protein, endotoxin, temperature, relative humidity, wind speed, and air pressure. For fine particles, the concentration of protein was positively correlated with that of particles (*r* = 0.654, *p* < 0.001) and endotoxin (*r* = 0.331, *p* = 0.003). For coarse particles, the concentration of protein was positively correlated with the concentration of particles (*r* = 0.357, *p* = 0.001) and endotoxin (*r* = 0.498, *p* < 0.001). The temperature was positively correlated with the coarse particle concentration and the concentrations of protein and endotoxin in coarse particles. Relative humidity was negatively correlated with the concentrations of fine particles and that of protein and endotoxin in fine particles. Air pressure was negatively correlated with the concentrations of protein and endotoxin in coarse particles.

**Table 3:** Correlation coefficient (*p* value) for environmental factors

	Fine particles	Protein (fine particles)	Endotoxin (fine particles)	Coarse particles	Protein (coarse particles)	Endotoxin (coarse particles)	Temperature	Relative humidity	Wind speed	Air pressure
Fine particles	1.00									
Protein (fine particles)	0.654 (< 0.001)	1.00								
Endotoxin (fine particles)	0.212 (0.064)	0.331 (0.003)	1.00							
Coarse particles	0.551 (< 0.001)	0.175 (0.128)	0.134 (0.246)	1.00						
Protein (coarse particles)	0.233 (0.041)	0.169 (0.142)	0.045 (0.695)	0.357 (0.001)	1.00					
Endotoxin (coarse particles)	− 0.104 (0.367)	− 0.085 (0.465)	0.474 (< 0.001)	0.187 (0.103)	0.498 (< 0.001)	1.00				
Temperature	0.015 (0.897)	− 0.045 (0.698)	0.218 (0.057)	0.244 (0.032)	0.592 (< 0.001)	0.633 (< 0.001)	1.00			
Relative humidity	− 0.548 (< 0.001)	− 0.516 (< 0.001)	− 0.523 (< 0.001)	− 0.187 (0.103)	0.015 (0.897)	− 0.006 (0.960)	− 0.033 (0.779)	1.00		
Wind speed	0.026 (0.822)	0.037 (0.750)	0.088 (0.445)	0.020 (0.860)	0.104 (0.366)	0.081 (0.485)	0.082 (0.476)	− 0.367 (0.001)	1.00	
Air pressure	0.043 (0.709)	0.176 (0.126)	− 0.102 (0.378)	− 0.135 (0.240)	− 0.567 (< 0.001)	− 0.463 (< 0.001)	− 0.511 (< 0.001)	0.008 (0.944)	− 0.294 (0.009)	1.00

Correlation coefficient (*p* value) was calculated with the weekly levels of airborne particles, protein, endotoxin, temperature, relative humidity, wind speed, and air pressure. Statistically significant, *p* < 0.05

### Association of environmental factor levels with emergency department visits for asthma

**Fig. 2:**
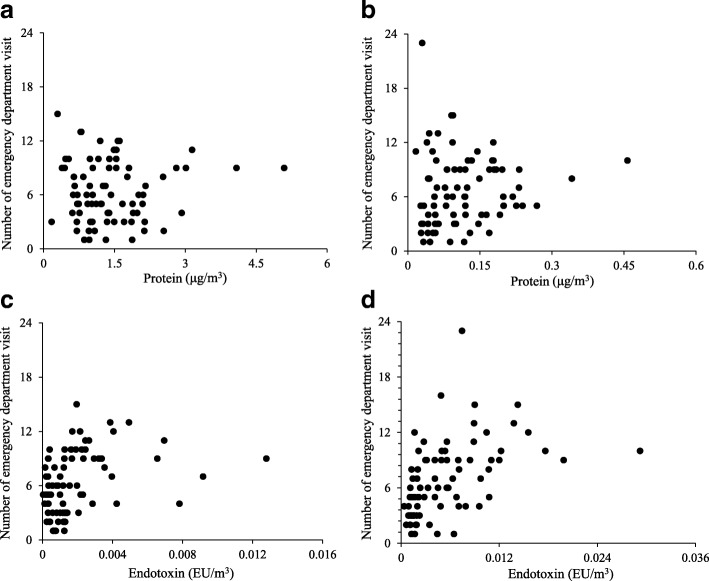
Scatter plots of protein and endotoxin levels with the number of emergency department visits for asthma. **a** Protein in fine particles. **b** Protein in coarse particles. **c** Endotoxin in fine particles. **d** Endotoxin in coarse particles

Figure 2 shows the scatter plots of protein and endotoxin levels with the number of emergency department visits. The influences of the concentrations of airborne particles, protein, and endotoxin in fine and coarse particles on the emergency department visits for asthma were evaluated by generalized linear models to fit a Poisson regression to adjust for meteorological factors (temperature, relative humidity, wind speed, and air pressure). As shown in Table 4, the concentrations of coarse particles and endotoxin in fine and coarse particles were statistically significant factors on emergency department visits for asthma. Among the meteorological factors, temperature was a significant factor in all cases. The concentrations of coarse particles and endotoxin in fine and coarse particles and temperature were positively associated with the number of emergency department visits for asthma.

**Table 4:** Results of Poisson regressions of air pollutants and meteorological factors on emergency department visits

Variables	Coef	SE	*p* value
Fine particles	− 0.009	0.011	0.414
Intercept	− 16.799	16.449	0.307
Temperature	0.046	0.010	< 0.001
Relative humidity	− 0.009	0.009	0.322
Wind speed	− 0.213	0.174	0.222
Air pressure	0.019	0.016	0.235
Protein (fine particles)	0.032	0.062	0.609
Intercept	− 16.566	16.538	0.317
Temperature	0.046	0.010	< 0.001
Relative humidity	− 0.002	0.009	0.823
Wind speed	− 0.170	0.172	0.324
Air pressure	0.018	0.016	0.264
Endotoxin (fine particles)	59.792	20.907	0.004
Intercept	− 18.420	16.674	0.269
Temperature	0.043	0.010	< 0.001
Relative humidity	0.009	0.009	0.333
Wind speed	− 0.100	0.170	0.556
Air pressure	0.019	0.016	0.242
Coarse particles	0.044	0.013	0.001
Intercept	− 17.309	16.523	0.295
Temperature	0.042	0.010	< 0.001
Relative humidity	− 0.001	0.008	0.944
Wind speed	− 0.172	0.171	0.314
Air pressure	0.018	0.016	0.251
Protein (coarse particles)	− 1.011	0.702	0.150
Intercept	− 12.459	16.738	0.457
Temperature	0.049	0.010	< 0.001
Relative humidity	− 0.004	0.007	0.588
Wind speed	− 0.186	0.168	0.270
Air pressure	0.014	0.016	0.383
Endotoxin (coarse particles)	29.637	9.044	0.001
Intercept	− 13.346	16.850	0.428
Temperature	0.030	0.011	0.007
Relative humidity	− 0.007	0.008	0.343
Wind speed	− 0.210	0.174	0.226
Air pressure	0.015	0.016	0.349

*Coef* regression coefficient, *SE* standard error of the regression

Statistically significant, *p* < 0.05

To consider the seasonal variation, we stratified the data by season and then analyzed the influences of environmental factors on the emergency department visits for asthma (Table 5). Coarse particle level was a statistically significant factor in spring and autumn and was positively associated with the emergency department visits for asthma. Protein level in fine particles was negatively associated with emergency department visits for asthma in summer. On the other hand, the protein level in coarse particles was a significantly positive factor in winter for emergency department visits. The endotoxin levels in both types of particles were not statistically significant in any season. Temperature was a significant factor in all cases in spring and in four of six cases in winter, and the association with emergency department visits was positive.

**Table 5:** Results of seasonal Poisson regressions of air pollutants and meteorological factors on emergency department visits

Variables	Spring			Summer			Autumn			Winter		
	Coef	SE	*p* value	Coef	SE	*p* value	Coef	SE	*p* value	Coef	SE	*p* value
Fine particles	0.024	0.018	0.199	− 0.082	0.055	0.131	0.052	0.032	0.107	0.003	0.032	0.931
Intercept	− 29.035	37.402	0.438	− 22.254	77.335	0.774	9.183	34.969	0.793	45.921	42.385	0.279
Temperature	0.088	0.033	0.007	0.040	0.045	0.375	0.038	0.032	0.239	0.107	0.052	0.039
Relative humidity	0.016	0.016	0.311	− 0.021	0.043	0.621	0.016	0.019	0.419	0.036	0.029	0.214
Wind speed	0.898	0.567	0.113	− 0.608	0.453	0.180	0.537	0.403	0.183	0.411	0.500	0.411
Air pressure	0.026	0.036	0.477	0.027	0.079	0.736	− 0.010	0.034	0.763	− 0.048	0.041	0.245
Protein (fine particles)	− 0.128	0.121	0.291	− 1.039	0.521	0.046	0.098	0.129	0.449	0.048	0.233	0.837
Intercept	− 48.966	38.962	0.209	− 53.432	80.320	0.506	11.460	34.557	0.740	49.651	46.418	0.285
Temperature	0.119	0.039	0.002	0.011	0.047	0.822	0.038	0.034	0.252	0.107	0.051	0.035
Relative humidity	< 0.001	0.015	0.974	0.001	0.030	0.977	− 0.001	0.015	0.966	0.035	0.028	0.221
Wind speed	0.632	0.544	0.246	− 0.505	0.394	0.200	0.262	0.359	0.465	0.367	0.502	0.465
Air pressure	0.047	0.038	0.210	0.057	0.082	0.488	− 0.010	0.034	0.756	− 0.051	0.045	0.254
Endotoxin (fine particles)	22.987	43.993	0.601	28.411	181.928	0.876	23.465	34.868	0.501	97.331	399.535	0.808
Intercept	− 38.240	37.175	0.304	33.846	70.257	0.630	6.580	34.906	0.850	49.851	45.574	0.274
Temperature	0.096	0.032	0.003	0.049	0.049	0.309	0.028	0.032	0.390	0.103	0.051	0.042
Relative humidity	0.010	0.015	0.517	0.035	0.028	0.210	0.004	0.020	0.835	0.044	0.046	0.336
Wind speed	0.732	0.552	0.185	− 0.163	0.412	0.692	0.333	0.382	0.382	0.361	0.501	0.471
Air pressure	0.036	0.036	0.317	− 0.035	0.071	0.618	− 0.006	0.034	0.864	− 0.052	0.045	0.248
Coarse particles	0.065	0.026	0.013	0.048	0.071	0.496	0.087	0.036	0.017	0.037	0.020	0.058
Intercept	− 40.870	37.408	0.275	42.374	70.477	0.548	− 2.956	35.372	0.933	34.113	42.135	0.418
Temperature	0.103	0.034	0.003	0.035	0.051	0.500	0.042	0.033	0.212	0.096	0.052	0.066
Relative humidity	0.014	0.015	0.349	0.033	0.028	0.227	0.007	0.015	0.647	0.042	0.029	0.146
Wind speed	0.909	0.555	0.101	− 0.155	0.330	0.640	0.185	0.358	0.606	0.631	0.496	0.203
Air pressure	0.037	0.036	0.299	− 0.044	0.071	0.537	0.003	0.034	0.933	− 0.037	0.041	0.361
Protein (coarse particles)	− 4.194	2.563	0.102	− 0.635	2.404	0.792	− 1.318	0.909	0.147	5.056	2.264	0.026
Intercept	− 6.415	42.314	0.879	29.896	71.928	0.678	13.038	33.558	0.698	41.434	41.091	0.313
Temperature	0.106	0.033	0.001	0.052	0.049	0.295	0.044	0.033	0.185	0.114	0.052	0.028
Relative humidity	0.002	0.014	0.867	0.038	0.030	0.216	< 0.001	0.015	0.983	0.036	0.028	0.205
Wind speed	0.609	0.551	0.269	− 0.165	0.359	0.646	0.256	0.355	0.471	0.361	0.467	0.440
Air pressure	0.006	0.041	0.892	− 0.031	0.072	0.663	− 0.012	0.033	0.716	− 0.044	0.040	0.272
Endotoxin (coarse particles)	9.069	32.095	0.778	45.832	26.900	0.088	10.432	15.789	0.509	311.199	208.761	0.136
Intercept	− 34.800	38.148	0.362	18.278	70.654	0.796	9.706	34.671	0.780	56.880	44.515	0.201
Temperature	0.094	0.035	0.007	0.019	0.049	0.706	0.018	0.037	0.619	0.059	0.058	0.314
Relative humidity	0.006	0.014	0.677	0.022	0.028	0.434	− 0.005	0.015	0.731	0.046	0.029	0.112
Wind speed	0.657	0.545	0.228	− 0.315	0.362	0.384	0.243	0.359	0.498	0.254	0.485	0.600
Air pressure	0.033	0.037	0.370	− 0.018	0.071	0.799	− 0.008	0.034	0.813	− 0.059	0.043	0.171

*Coef* regression coefficient, *SE* standard error of the regression

Statistically significant, *p* < 0.05

## Discussion

In this study, we collected fine and coarse airborne particles in Kyoto City for 77 weeks (21 months), analyzed protein and endotoxin concentrations, and investigated the association of these concentrations, temperature, relative humidity, wind speed, and air pressure with the number of emergency department visits for asthma. We found that the concentrations of coarse particles and endotoxin in fine and coarse particles were significantly positively correlated with the number of emergency department visits in a generalized linear model to fit a Poisson regression to adjust for meteorological factors (Table 4). To our knowledge, this is the first report to show that atmospheric endotoxin is significantly associated with asthma exacerbation in Japan.

Many studies have examined the relationship between exposure to fine and coarse particles and asthma, but their results have not been consistent. Some studies have reported a significant association between exposure to these particles and an increased risk of clinic visits and hospitalization for asthma, whereas others have found no significant association [1, 2, 5]. These results suggest that the constituents of airborne particles cause the exacerbation of asthma and that diversity among these constituents may result in inconsistencies regarding the health effects. In the present study, the concentrations of airborne fine particles and protein in particles were not significantly associated with the number of emergency department visits for asthma in the case of the whole study period (Table 4). Asthma is an allergic disease, and there are several reports on allergic proteins originating from pollen and fungi in outdoor air [6, 7]. In addition, various materials such as animal dander, as well as activities such as the combustion of organic materials, are also potential sources of protein in outdoor air [8, 10, 21, 22]. Kang et al. analyzed the protein and other constituents in airborne particles collected in Hefei, China, and concluded that anthropogenic activities and biomass combustion were the main sources of protein in the outdoor air in the study area [8]. In a previous study, we found that the concentration of protein in fine particles was higher than that in coarse particles in Sasebo, Nagasaki, Japan, and that the protein concentration was positively associated with the concentrations of combustion products (nitrate and sulfate ions), indicating that the combustion of organic materials may be a source of protein. In this study, the concentration of protein in fine particles was higher than that in coarse particles collected in Kyoto (Additional file 1: Figure S2). These results suggest that the protein detected in this study may have originated from the combustion of organic materials and that the chemical structures of the proteins were altered by heat and photochemical reactions during transportation in the atmosphere and had lost their biological activity.

In this study, endotoxins in fine and coarse particles were significantly correlated with the number of emergency department visits for asthma. Endotoxin inhalation in a challenge setting induced the hallmarks of asthma, bronchoconstriction, airway inflammation, and bronchial hyper-responsiveness [23, 24]. The Institute of Medicine reviewed articles published from 2000 to 2013 on indoor environmental exposure and exacerbation of asthma, concluding that there was sufficient evidence of an association between indoor exposure to endotoxins and the exacerbation of asthma [25]. However, the association between endotoxin in outdoor air and the exacerbation of asthma is unclear. Endotoxins are the major component of the outer membrane of Gram-negative bacteria. Park et al. reported that the abundance of bacteria on airborne particles increases on Asian dust days in Osaka, Japan, and that several classes of Gram-negative bacteria are dominant [26]. Asian dust are soil particles that are carried eastward by winds from arid and semiarid areas of northern China and Mongolia. Asian dust events are mainly observed in the spring and autumn in Japan [27]. Ichinose et al. detected endotoxins in soil samples collected from Asian dust source regions [28]. In this study period, Asian dust events were found on 6 days in February, March, April, and June by Japan Meteorological Agency, and endotoxin levels in particles collected for weeks including these days were high. In addition, high levels of endotoxin were detected in particles collected in autumn (Additional file 1: Figure S3). Further investigation is needed to determine the exact sources of endotoxins in airborne particles.

In the analysis considering the effects of seasonality (Table 5), the association of protein on emergency department visits for asthma was inconclusive; the regression coefficient of protein in fine particles was negative in summer, but that in coarse particles was positive in winter. To our knowledge, there were no reports on the atmospheric protein that improve asthmatic symptoms. The number of data in each season was small (12–22), and the inconsistency of the effects of protein may be caused by the insufficiency of data. Further sampling and analysis are necessary to confirm seasonal variation.

This study has several limitations. First, because we chose emergency department visits for asthma as a surrogate measurement for asthma exacerbation, the patients in this study may have had moderate to severe symptoms. This deviation of patients may have affected the association between environmental factors (particles, protein, and endotoxin) and the increased risk of asthma exacerbation. Second, we measured weekly levels of environmental factors and examined the association of these factors with emergency department visits for asthma, because the purpose of this study was to clarify the association for long term and daily measurement of these environmental factors was too laborious for long-term study. However, the levels of environmental factors may fluctuate every day. Therefore, further investigation using daily data should also be performed in the future. Third, we were unable to measure the effect of indoor endotoxin level on the exacerbation of asthma in this study. However, because endotoxin concentrations in indoor air are significantly correlated with those in outdoor air [29], we believe that the endotoxin concentration of outdoor air is a suitable surrogate for endotoxin in the environment in order to analyze its association with health effects in a large population study. Fourth, data were available for 21 months in this study; thus, more long-term study is necessary to clarify the seasonal patterns.

## Conclusion

In this study, we found that atmospheric endotoxin was a significant factor on emergency department visits for asthma even after adjusting for meteorological factors. Endotoxin level was positively associated with the number of emergency department visits for asthma. In contrast, the association between atmospheric protein and asthma exacerbation was inconclusive. This is the first report on the association of atmospheric endotoxin and protein with asthma exacerbation. Further study is necessary to ascertain the effects of atmospheric endotoxin and protein on asthma exacerbation.

## Additional file

Additional file 1: **Figure S1.** Mass concentrations of fine (a) and coarse (b) particles in the outdoor air of Kyoto, Japan (September 2014–May 2016). Figure S2. Concentrations of protein in fine (a) and coarse (b) particles in the outdoor air of Kyoto, Japan (September 2014–May 2016). Figure S3. Concentrations of endotoxin in fine (a) and coarse (b) particles in the outdoor air of Kyoto, Japan (September 2014–May 2016). (ZIP 1803 kb)

## Abbreviations

LAL: Limulus amebocyte lysate
PM_2.5_: Particulate matter ≤ 2.5 μm
