# Supplementary material for: Association of airborne particles, protein, and endotoxin with emergency department visits for asthma in Kyoto, Japan
Source: Environ Health Prev Med. 2018 Aug 28;23:41. doi: 10.1186/s12199-018-0731-2 (PMC6114267; doi:10.1186/s12199-018-0731-2)
Supplement: Supplementary file 1 — Figure S1. Mass concentrations of fine (a) and coarse (b) particles in the outdoor air of Kyoto, Japan (September 2014–May 2016). Figure S2. Concentrations of protein in fine (a) and coarse (b) particles in the outdoor air of Kyoto, Japan (September 2014–May 2016). Figure S3. Concentrations of endotoxin in fine (a) and coarse (b) particles in the outdoor air of Kyoto, Japan (September 2014–May 2016). (ZIP 1803 kb) [file 12199_2018_731_MOESM1_ESM.zip › renamed_d7e1f.pptx]

## Slide 1
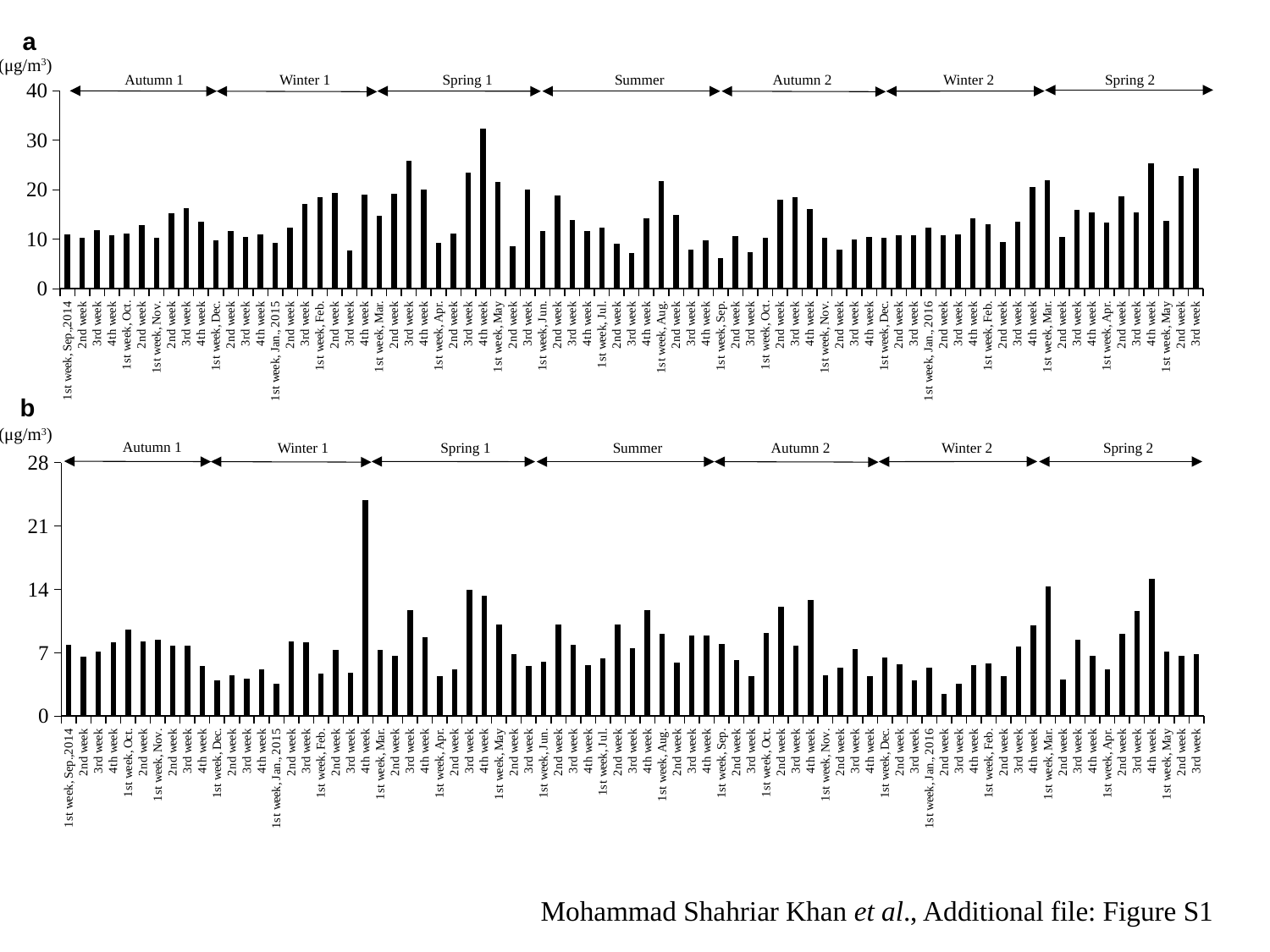

a
### Chart
| Category | |
|---|---|
| 1st week, Sep.,2014 | 10.813706621163119 |
| 2nd week | 10.119248397785672 |
| 3rd week | 11.805789797416615 |
| 4th week | 10.714498303537768 |
| 1st week, Oct. | 11.11133157403917 |
| 2nd week | 12.698664656044764 |
| 1st week, Nov. | 10.21845671541102 |
| 2nd week | 15.079664279053157 |
| 3rd week | 16.170955772931983 |
| 4th week | 13.393122879422235 |
| 1st week, Dec. | 9.722415127284258 |
| 2nd week | 11.508164844540577 |
| 3rd week | 10.416873350661762 |
| 4th week | 10.813706621163119 |
| 1st week, Jan., 2015 | 9.127074673359862 |
| 2nd week | 12.301831385543377 |
| 3rd week | 17.063830631560123 |
| 1st week, Feb. | 18.353538760689705 |
| 2nd week | 19.34562193694322 |
| 3rd week | 7.5398321395265855 |
| 4th week | 18.94878866644182 |
| 1st week, Mar. | 14.58362269092643 |
| 2nd week | 19.04799698406716 |
| 3rd week | 25.79416258259095 |
| 4th week | 19.940871842695298 |
| 1st week, Apr. | 9.127165221532183 |
| 2nd week | 11.111331574039134 |
| 3rd week | 23.41316295958251 |
| 4th week | 32.34191154586406 |
| 1st week, May | 21.428996607075515 |
| 2nd week | 8.432706998154723 |
| 3rd week | 19.940871842695255 |
| 1st week, Jun. | 11.508164844540577 |
| 2nd week | 18.75037203119106 |
| 3rd week | 13.789956149923633 |
| 4th week | 11.508164844540577 |
| 1st week, Jul. | 12.30183138554333 |
| 2nd week | 9.062564058302504 |
| 3rd week | 7.1429988690251855 |
| 4th week | 14.18678942042503 |
| 1st week, Aug. | 21.627413242326238 |
| 2nd week | 14.88124764380245 |
| 3rd week | 7.738248774777306 |
| 4th week | 9.623206809658921 |
| 1st week, Sep. | 6.150915692771683 |
| 2nd week | 10.51608166828707 |
| 3rd week | 7.341415504275863 |
| 1st week, Oct. | 10.218456715410996 |
| 2nd week | 17.95670549018826 |
| 3rd week | 18.353538760689705 |
| 4th week | 15.972539137681308 |
| 1st week, Nov. | 10.119248397785658 |
| 2nd week | 7.738248774777306 |
| 3rd week | 9.920831762534979 |
| 4th week | 10.317665033036379 |
| 1st week, Dec. | 10.218355340826815 |
| 2nd week | 10.615289985912396 |
| 3rd week | 10.615289985912396 |
| 1st week, Jan., 2016 | 12.202623067918038 |
| 2nd week | 10.615184674451111 |
| 3rd week | 10.813706621163119 |
| 4th week | 14.087581102799652 |
| 1st week, Feb. | 12.933531992384736 |
| 2nd week | 9.325581856782904 |
| 3rd week | 13.492331197047573 |
| 4th week | 20.436913430822038 |
| 1st week, Mar. | 21.82582987757691 |
| 2nd week | 10.317665033036379 |
| 3rd week | 15.774122502430584 |
| 4th week | 15.377289231929232 |
| 1st week, Apr. | 13.194706244171511 |
| 2nd week | 18.55195539594038 |
| 3rd week | 15.278080914303848 |
| 4th week | 25.298120994464167 |
| 1st week, May | 13.690612010039816 |
| 2nd week | 22.718704736205087 |
| 3rd week | 24.30603781821068 |(μg/m3)
Autumn 1
Summer
Autumn 2
Spring 1
Winter 2
Winter 1
Spring 2
b
### Chart
| Category | |
|---|---|
| 1st week, Sep.,2014 | 7.837457092402628 |
| 2nd week | 6.547748963273081 |
| 3rd week | 7.043790551399829 |
| 4th week | 8.135082045278677 |
| 1st week, Oct. | 9.523998492033572 |
| 2nd week | 8.234290362904026 |
| 1st week, Nov. | 8.432706998154725 |
| 2nd week | 7.738248774777285 |
| 3rd week | 7.738248774777285 |
| 4th week | 5.456457469394228 |
| 1st week, Dec. | 3.869124387388631 |
| 2nd week | 4.464374293140752 |
| 3rd week | 4.067541022639353 |
| 4th week | 5.158832516518189 |
| 1st week, Jan., 2015 | 3.5714640026190767 |
| 2nd week | 8.234290362904023 |
| 3rd week | 8.135082045278685 |
| 1st week, Feb. | 4.66279092839143 |
| 2nd week | 7.242207186650526 |
| 3rd week | 4.76199924601679 |
| 4th week | 23.809996230083932 |
| 1st week, Mar. | 7.242207186650546 |
| 2nd week | 6.646957280898425 |
| 3rd week | 11.706581479791277 |
| 4th week | 8.631123633405423 |
| 1st week, Apr. | 4.365165975515391 |
| 2nd week | 5.158832516518189 |
| 3rd week | 13.88916446754896 |
| 4th week | 13.29391456179685 |
| 1st week, May | 10.11924839778568 |
| 2nd week | 6.845373916149125 |
| 3rd week | 5.456457469394228 |
| 1st week, Jun. | 5.952499057520966 |
| 2nd week | 10.11924839778568 |
| 3rd week | 7.837457092402623 |
| 4th week | 5.555665787019567 |
| 1st week, Jul. | 6.3493323280223875 |
| 2nd week | 10.03355306454919 |
| 3rd week | 7.440623821901225 |
| 4th week | 11.706581479791277 |
| 1st week, Aug. | 9.027956903906821 |
| 2nd week | 5.853290739895628 |
| 3rd week | 8.829540268656121 |
| 4th week | 8.829540268656121 |
| 1st week, Sep. | 7.936665410027977 |
| 2nd week | 6.150915692771683 |
| 3rd week | 4.365165975515391 |
| 1st week, Oct. | 9.127165221532183 |
| 2nd week | 12.004206432667315 |
| 3rd week | 7.738248774777262 |
| 4th week | 12.797872973670113 |
| 1st week, Nov. | 4.464374293140731 |
| 2nd week | 5.258040834143529 |
| 3rd week | 7.341415504275863 |
| 4th week | 4.365165975515369 |
| 1st week, Dec. | 6.448476671395544 |
| 2nd week | 5.654874104644928 |
| 3rd week | 3.869124387388631 |
| 1st week, Jan., 2016 | 5.258040834143529 |
| 2nd week | 2.3809760017460513 |
| 3rd week | 3.5714994345125928 |
| 4th week | 5.5556657870195885 |
| 1st week, Feb. | 5.794222332588345 |
| 2nd week | 4.365165975515369 |
| 3rd week | 7.639040457151924 |
| 4th week | 10.020040080160319 |
| 1st week, Mar. | 14.28599773805035 |
| 2nd week | 3.9683327050139705 |
| 3rd week | 8.432706998154723 |
| 4th week | 6.646957280898425 |
| 1st week, Apr. | 5.158832516518189 |
| 2nd week | 9.027956903906821 |
| 3rd week | 11.607373162165917 |
| 4th week | 15.079664279053148 |
| 1st week, May | 7.043720671832058 |
| 2nd week | 6.64695728089843 |
| 3rd week | 6.84537391614913 |(μg/m3)
Autumn 1
Summer
Autumn 2
Spring 1
Winter 2
Winter 1
Spring 2
Mohammad Shahriar Khan et al., Additional file: Figure S1
